# Immunometabolic interactions in individuals with down syndrome across childhood, adolescence and adulthood in relation to their siblings

**DOI:** 10.3389/fimmu.2026.1838695

**Published:** 2026-07-07

**Authors:** Anna Tylutka, Agnieszka Zembron-Lacny, Marta Hetman, Aleksandra Bodetko, Helena Moreira, Ewa Barg

**Affiliations:** 1 Department of Applied and Clinical Physiology, Collegium Medicum University of Zielona Gora, Zielona Gora, Poland; 2 Department of Pediatric Bone Marrow Transplantation, Oncology and Hematology, Wroclaw Medical University, Wroclaw, Poland; 3 Department of Basic Medical Sciences Faculty of Pharmacy Wroclaw Medical University, Wroclaw, Poland

**Keywords:** cytokine, down syndrome, inflammation, lipid profile, older, young

## Abstract

**Introduction and aim:**

Down syndrome is the most common chromosomal disorder characterized by a wide spectrum of clinical symptoms such as immune system dysregulation and co-occurring metabolic disorders, including an increased risk of cardiovascular disease. Therefore, the aim of this study was to evaluate the immunometabolic interactions in children adolescents and adults with Down syndrome (DS) and to compare selected inflammatory and metabolic parameters with those observed in their siblings.

**Materials and methods:**

The study included n= 63 individuals who were divided into two groups: group with DS n=42 (mean age: 14.2 ± 6.6) and control group (CG) n=21 (mean age: 15.6 ± 6.9). In addition, patients in both groups were also divided according to age ≤ 18 years and >18 years of age. Carbohydrate-lipid and immunological profiles were analyzed using spectrophotometric and immunoenzymatic methods. Statistical analysis was performed using R studio software.

**Results:**

In the DS group ≤ 18 years significantly higher obesity rates, i.e., Ponderal Mass Index (TMI), were observed (p=0.04), which was also associated with statistically significantly higher level of non-HDL (p=0.02) and apoB (p=0.04). Among lipid parameters, apolipoprotein A demonstrated relatively high diagnostic utility (AUC = 0.818, sens%=60.0, spec%=95.2). Significantly lower cytokine levels were observed in the DS group for IL-10 (p=0.006), IL-13 (p<0.01), and IL-22 (p=0.002). The highest diagnostic utility among the assessed cytokines was demonstrated for IL-5 (AUC = 0.814, sens%=71.10, spec%=88.1).

**Conclusion:**

The analyses conducted indicate significant differences between the studied groups of patients with Down syndrome and the control group. The presence of an additional copy of 21 the chromosome leads to changes in the immune system, influences the heterogeneous cytokine profile, and consequently may increase the development of metabolic disorders.

## 1 Introduction

Down syndrome (DS, Trisomy 21, T21) is the most common chromosomal disorder, with a global incidence of approximately 1:1000–1100 live births (1). The presence of an additional chromosome 21, or at least part of it, causes chronic and heightened inflammation described as an inflammatory phenotype comparable to, and even exceeding, that of intensive care patients. This persistent inflammation, driven by genetic overexpression, contributes to accelerated immunoaging, and therefore susceptibility to hematologic, endocrine, cardiovascular neurodegenerative and autoimmune diseases (2, 3). Advances in medicine have significantly increased the average life expectancy of individuals with DS to over 50 years, which has brought new health challenges in this population (4). Despite substantial progress in DS research, many aspects of DS remain insufficiently explored, and the diversity of symptoms and clinical correlations complicates diagnostic evaluation. Patients with DS (PWDS) are genetically predisposed to metabolic disturbances that are closely linked to the regulation of cytokine dysregulation (3, 5, 6). Malle et al. (7) demonstrated that enhanced cytokine dysregulation in DS is correlated with greater clinical immune dysfunction, specifically higher autoantibody production and autoimmune susceptibility. Important genes like the autoimmune regulator (AIRE) gene and four of the six interferon receptor subunits (which act as receptors for both type III interferon (IFN) ligands and cytokines interleukin-10, interleukin-22 and interleukin-26) are found on this chromosome, leading to an unbalanced gene dosage in individuals with DS (8, 9). Also, human leukocyte antigen (HLA) subtyping studies have shown that the major histocompatibility complex class II (MHC II) allele DQA1 0301, which is controlled by immune regulatory genes on chromosome 21, is strongly overrepresented in individuals with Down syndrome and autoimmune thyroid disease (10).

PWDS also exhibit a marked tendency toward obesity, driven by a positive energy balance; adults with DS have twice the likelihood of obesity and nearly four times the likelihood of extreme obesity compared with adults without DS (11). In addition, PWDS tend to lead less active lifestyles, which accelerates metabolic disturbances. The nature of lipid metabolism abnormalities in DS remains unclear, with limited, inconsistent, and often contradictory data. From a physiological perspective, PWDS appear simultaneously predisposed to, and protected from, dyslipidemia and its consequences (12). A meta-analysis published by Guerrero et al. (13) in 2024 summarized the results of studies from the last 30 years on serum lipid levels in people with Down syndrome. The analysis, including n=671 people with DS and n=898 healthy individuals, indicated significant differences in the levels of total cholesterol (p< 0.0001), high density lipoprotein cholesterol (HDL-C) (p = 0.05), and triglycerides (p= 0.0005). Changes in the lipid profile in individuals with DS may be associated with a higher incidence of overweight and obesity, diabetes, or a different lifestyle. An altered lipid profile is also associated with an increased incidence of atherosclerotic lesions in individuals with DS. Elevated low-density lipoprotein cholesterol (LDL-C) promotes plaque formation, whereas HDL-C supports cholesterol clearance. Increased homocysteine (HCY) is considered an additional risk factor for cardiovascular diseases (CVD) (14). Lipoprotein(a) (Lp(a)) may further elevate CVD risk, and its largely genetic determination makes it difficult to modify through lifestyle measures (15). Incorporating Lp(a) into overall cardiovascular risk assessment is therefore clinically relevant. Observational studies conducted by Pedersen et al. (16) - the median age at the end of the observation period was 35 years in the DS population and 42 years in the comparison group showed that the DS group was associated with an increased risk of not only ischemic stroke (hazart ratio; HR) 4.41, but also hemorrhagic stroke (HR) 5.14. reports point to the significant need to test lipid parameters in children with DS, which will enable earlier detection of the disorder and, if necessary, enable the implementation of treatment, reducing mortality. DS is also associated with distinct immunological and metabolic alterations. Interactions among genetic factors, metabolites, and cytokine expression contribute to coordinated regulation of metabolic pathways and immune responses (6, 17). A meta-analysis by Zhang et al. (18) demonstrated significantly elevated pro-inflammatory markers—IL-1β, TNF-α, and IFN-γ—in children with DS. Cytokines such as IL17, IFN-γ, TNF-α, and anti-inflammatory IL-10 are secreted by T cells in the event of autoimmunity or infection (19). There is also evidence that IL-10, a cytokine that serves to suppress the inflammatory response, is elevated in individuals with DS, raising the hypothesis that pronounced anti-inflammatory signals may be responsible for the increased incidence of respiratory and pneumococcal infections (19). The contribution of other cytokines, such as IL-22 or IL-2, to DS pathophysiology remains unclear. However, work by Gerez et al. (20) showed selective impairment of IL-2 gene expression in peripheral blood mononuclear cells from children with DS, suggesting altered T-cell activation and, consequently, dysregulated immune responses (20). Therefore the aim of this study was to investigate carbohydrate and lipid metabolism parameters in relation to the inflammatory phenotype in individuals with Down syndrome compared with their healthy siblings as a control group, with additional analyses performed according to age stratification (≤ 18 years and >18 years) to assess potential age-dependent differences in immunometabolic interactions.

## 2 Materials and methods

### 2.1 Study design and participants

The participants consisted of a study group of 42 children and adolescents with DS (group with DS); 17 females; mean age of 14.2 ± 6.6 old and the control group (CG) of 21 siblings of the individuals with DS (10 females; mean age of 15.6 ± 6.9 years old). In the DS group, we had: n=29 children ≤ 18 years of age and n=13 individuals >18 years of age. In the control group, n=13 individuals were ≤ 18 years of age and n=8 individuals were > 18 years of age. The research was conducted in accordance with the Declaration of Helsinki and received approval from the Bioethics Committee at Wroclaw Medical University (KB 674/2020). Eligibility criteria for the DS group required a confirmed diagnosis of DS based on genetic testing; for the control group, eligibility was limited to being a sibling of a participant with DS. During recruitment, no acute infections were observed in DS group. The medications taken by the patients ≤ 18 years of age in the DS group was only L-thyroxine (100%) and over > 18 years of age it was also 100% L-thyroxine. The group of patients with Down’s syndrome studied had been under the care of an endocrinologist since infancy. The control group had no history of chronic diseases, including autoimmune diseases, or acute infections. Written informed consent was obtained from parents or legal guardians prior to participation, and anthropometric assessments were performed thereafter. Administrative permissions were secured from all participating institutions to allow access to relevant patient data.

### 2.2 Data collection

Participants were recruited from multiple regions of Poland. After obtaining written informed consent from parents or legal guardians, the enrolled children, adolescents and adults underwent anthropometric assessment and blood sampling at a pediatric clinic in Wroclaw, Poland.

### 2.3 Anthropometric analyses

The methodology and procedures for anthropometric measurements have been detailed in our prior research (21). BMI Standard Deviation Score (SDS) and Tri-Ponderal Mass Index (TMI) Calculation BMI was converted into SDS in line with our previously outlined methodologies (22). The Tri-Ponderal Mass Index was calculated according to the methodology described by Peterson et al. (23). For this study, the threshold to determine elevated TMI was set at the 95^th^ percentile – specifically, values surpassing 18.8 kg/m^3^ were considered elevated for boys, while for girls, the 95^th^ percentile value was set at 19.7 kg/m^3^ (23). Before proceeding with the TMI analysis, participants aged below 7 years were excluded from the dataset. This led to the exclusion of nine individuals: 6 from the DS and 3 from the control group.

BMI SDS values were calculated using the following formula:


x SDS=x value−x value for 50th centile/0,5x (x value for 50th centile−x value for 3rd centile) x−height/weight2

The reference range was -1.66 to +1.66. BMI SDS values, below -1.66 classify the patient as underweight. A range of +1.66 to 1.88 is considered overweight, and values above 1.88 are considered obese.

### 2.4 Peripheral blood samples

Blood samples from the patients were taken in the morning from the median cubital vein using S-Monovette-EDTA K_2_ tubes (Sarstedt, Austria) for morphology and S-Monovette - serum tubes were used for other biochemical and immunological markers. The patients were fasting 12 hours before the examination. Serum samples were left to clot for 45 min before centrifugation and then centrifuged at 3000 g for 10 min. Aliquots of serum were stored at -80 °C until testing.

### 2.5 Hematological variables

The assessment of peripheral blood morphology was assessed in the red blood cell system: red blood cells (RBC), hemoglobin (Hb), hematocrit (HCT), Mean Corpuscular Volume (MCV) and White Blood Cell System: Leukocytes (LEU), Lymphocytes (LYMPH), Neutrophils (NEU), Eosinophils (EOSO) and Basophils (BASO) using Sysmex XN1000.

### 2.6 Biochemical and hormonal parameters

All laboratory parameters were determined under fasting conditions. For lipid profile routine laboratory methods were used. Apolipoprotein A1 (ApoA1) and Apolipoprotein B (ApoB) were measured by immunotubidimetric method with goat anti-human apolipoprotein A1 antibody and anti-human apolipoprotein B antibody, respectively, whereas for Lp(a) determination particle enhanced immunoturbidimetric test was applied. Homocystein was measured with Diazyme Laboratories, Poway, USA reagent kit on the Konelab 20i analyser (ThermoScientific, Vantta, Finland). Homeostatic Model Assessment Insulin Resistance (HOMA-IR) was calculated according to Sitar-Tăut et al. (24), HOMA-IR = insulin (μU/mL) × glycaemia (mg/dL)/405. The assessment of other biochemical parameters, including albumin, lactate dehydrogenase (LDH) and renal parameters: urea, creatinine, was performed using the BM200 automatic analyzer from Biomaxima (Lublin, Poland). Due to the higher frequency of autoimmune diseases in the thyroid area, thyroid function was assessed using: thyroid stimulating factor (TSH) and free thyroxine (fT_4_).

### 2.7 Immunological variables

In order to assess the function of the immune system, ELISA kits from SunRed Biotechnology Company (Shanghai, China) method was used to assess pro and anti-inflammatory cytokines: IL-5 (sensivity: 1.5 pg/mL), IL-13 (sensivity: 0.7 pg/mL), IL-22 (sensivity: 0.722ng/L) IL-10 (sensivity 1.142 pg/ml), as well as IL-33 (sensivity: 0.573 ng/L) and IL-2 which are attributed to both pro-inflammatory and anti-inflammatory functions. C reactive protein (CRP) was measured using a high-sensitivity assay in duplicate by means of a commercial kit from DRG International (USA) with a detection limit of 0.001.

### 2.8 Statistical analysis

All statistical analyses were performed using R system 4.2.1 (25) (accessed Jan 2 2026). The variables were reported as mean values ± standard deviation (SD). The assumptions for the use of parametric or nonparametric tests were checked using the Shapiro-Wilk tests to evaluate the normality of the distributions. The significant differences in mean values between the groups were assessed by the one-way ANOVA. If the normality and homogeneity assumptions were violated, the Mann -Whitney nonparametric test was used. Spearman rank correlation coefficient (r_s_) or Pearson correlation coefficient (R) was used Spearman rank correlation coefficient (rs) or Pearson correlation coefficient (R) was used to analyse the correlation. The optimal threshold value for clinical stratification (cut-off value) was obtained by calculating the Youden index. Diagnostic performance was evaluated using Receiver Operating Characteristic (ROC) curve analysis implemented in R. ROC analysis assesses the ability of a continuous variable or predictive model to discriminate between two outcome classes across all possible threshold values. The ROC curve was generated by plotting sensitivity (true positive rate) against 1 − specificity (false positive rate) for a range of cut-off points. The overall discriminatory capacity of the variable/model was quantified using the Area Under the Curve (AUC), where an AUC of 0.5 indicates no discriminative ability and an AUC of 1.0 indicates perfect discrimination. The optimal cut-off value was determined using the Youden index. The threshold maximizing the Youden index was selected as the optimal cut-off because it provides the best balance between sensitivity and specificity. Statistical significance was set at p<0.05 and 95% confidence interval.

## 3 Results

### 3.1 Body composition and basic characteristics of the group

In the Down syndrome group, the mean age of the study participants was 14.2 ± 6.6. The patients with DS were significantly shorter than the control group (p=0.037). There were no statistically differences between group >18 years of age in BMI index (p= 0.241). The analysis of body weight in children, i.e. ≤ 18 years of age, was performed using the BMI SDS and TMI indicators. No statistical significance was observed for the BMI SDS index (p=0.557) and only for the TMI index (p=0.04).Statistical significance between groups was also noted in systolic blood pressure (p=0.023), it was lower in DS group (**Table 1**).

**Table 1:** Basic characteristics of the study group.

Variables	Group with DS n=42	Control group n=21	p-value
	mean ± SD	mean ± SD	
Age [years]	14.2 ± 6.6	15.6 ± 6.9	0.049
Body mass [kg]	44.6 ± 19.1	48.5 ± 18.8	0.512
Height [cm]	1.4 ± 0.2	1.5 ± 0.2	0.037
BMI [kg/m^2^] > 18 y.o.	25.3± 6.2	22.7 ± 2.5	0.241
BMI SDS ≤ 18 y.o.	1.1± 1.21	0.8± 1.2	0.557
TMI [kg/m3] <18 y.o.	15.8 ± 2.9	13.9 ± 1.6	0.04
RR systolic	103.2 ± 21.1	114.4 ± 8.8	0.023
RR diastolic	66.4 ± 15.4	71.4 ± 14.1	0.115
Pulse [ud/min]	89.6 ± 90.9	79.7 ± 10.7	0.346

BMI, body mass index; SDS, Standard Deviation Score; TMI, Tri-Ponderal Mass Index.

### 3.2 Hematological variables

Evaluation of red blood cell parameters showed that in the younger children aged 5–10 years with DS (n=4), has Hb was within the reference range. In the older group, only two individuals in group with DS demonstrated hemoglobin levels above the reference range. In the control group, all Hb results were within the reference range. A correlation trend was shown between body weight and hemoglobin (rs=0.408, p<0.01). In turn MCV shows higher mean values in individuals with DS 92.8 ± 4.7 compared to the control group 85.2 ± 4.9 (**Table 2**). Analysis of the white blood cell system showed that all assessed parameters were within the reference range. However, for leukocytes and lymphocytes, we observed significantly higher values in the control group compared to the group with DS: p = 0.047 for leukocytes and p = 0.034 for lymphocytes, respectively.

**Table 2:** Hematological variables.

Variables	Reference values	Group with DS n=42	Control group n=21	p-value
		mean ± SD	mean ± SD	
RBC [10^6^/µl]	child 3.8 – 5.2 Adults Women 4,.0 – 5.2 Men - 4.4 – 5.7	4.6 ± 0.4	4.6 ± 0.3	0.701
Hb [g/dl]	Child 5–10 yo 10.9 – 14.9 10–15 yo 11.4 – 15.4 Adults Women 11.8 – 15.2 Men - 3.4 – 17.0	14.7 ± 1.4	13.2 ± 0.7	<0.001
HCT [%]	Child 2–6 yo 34 – 40 7–12 yo 35 – 45 Adults Women 37 – 46 Men -37 – 46	42.9 ± 3.9	39.1 ± 0.2	<0.001
MCV [fl]	Child 5–7 yo 76 – 96 8–15 yo 78 - 98 Adults 82.5 – 97.4	92.8 ± 4.7	85.2 ± 4.9	<0.001
Leukocytes [10^3^/µl]	Child 4.5 – 13.5 Aduls 3.7 – 9.2	6.1 ± 2.1	6.9 ± 1.4	0.047
Neutrophils [10^3^/µl]	Child 7–10 yo 1,5 – 8,5 11–16 yo 1.8 – 8.0 Adults 1.6 – 5.8	3.3 ± 1.8	3.5 ± 1.1	0.377
Lymphocytes [10^3/^µl]	Child 2–6 yo 1.5 – 7.0 6–12 yo 0.9 – 3.4 12–16 yo 1.2 – 5.2 Adults 1.1 – 3.3	3.0 ± 5.4	4.1 ± 2.7	0.034
Monocytes [10^3/^µl]]	Child 2–6 yo 0.19 – 0.94 6–12 yo 0.19 – 0.85 12–18 yo 0.18 – 0.78 Adults 0.3 – 0.8	0.8 ± 1.5	0.5 ± 0.1	0.712
Eosinophils [tys./ul]	Child 2–6 yo 0.03 – 0.53 7–12 yo 0.03 – 0.52 12–18 yo 0.02 – 0.38 Adults 0.05 – 0.53	0.1 ± 0.0	0.1 ± 0.1	0.010
Basophils [tys./ul]	Child 0.5–12 yo 0.01 – 0.06 12–18 yo 0.01 – 0.05 Adults 0.02 – 0.1	0.1 ± 0.0	0.0 ± 0.0	<0.001
Lymphocytes [%]	Child 2–6 yo 14.3 – 50.6 7–12 yo 13.1 – 45.2 13–18 yo 12.9 – 40.6 Adults 0.02 – 0.1	36.5 ± 9.8	39.1 ± 10.0	0.896
Neutrophils [%]	Child 2–12 yo 32.3 – 76.9 13–18 lat 41.9 – 77.8 Adults 37.6 – 69.3	52.5 ± 10.4	50.8 ± 10.6	0.607
Monocytes [%]	Child 2–6 yo 4.1 – 12.2 6–12 yo 4.2 – 12.3 12–18 yo 4.1 – 12.3 Adults 5.3 – 12.4	8.2 ± 2.4	7.5 ± 1.0	0.329
Eosinophils [%]	Child 2–6 yo 0.0 – 4.1 6–12 yo 0.0 – 4.7 12–18 yo 0.0 – 4.0 Adults 0.9 – 8.3	1.3 ± 0.8	1.8. ± 0.9	0.065
Basophilse [%]	Child 2–6 yo 0.03 – 0.53 6–12 yo 0.03 – 0.52 12–18 yo 0.02 – 0.38 Adults 0.3 – 1.6	0.2 ± 0.2	0.6 ± 0.2	<0.001

RBC, red blood cells; Hb, hemoglobin; HCT, hematocrit; MCV, mean corpuscular volume.

### 3.3 Biochemical variables

Lipid profile results showed statistical significance between the groups for LDL-C (p= 0.028). In the group with DS, 31% of individuals had LDL-C values above 110 mg/dl, and in the case of individuals in control group, only one had an LDL-C range above 110 mg/dl (129 mg/dl). Statistically higher HDL-C values were observed in the control group (59.2 ± 9.6) compared to group with DS (47.0 ± 10.2). In the DS group vs. the control group ≤ 18 years of age, statistically significant differences were observed for HDL-C (p= 0.01), non-HDL (p= 0.02), and apoB (p= 0.04). In the >18 years of age group, significant differences were also observed for HDL-C (p= 0.02) and also for apoA1 (p<0.001). Interestingly, the assessment of atherogenic Lp (a) did not show any significance between the studied groups. Neither glucose nor insulin showed statistical significance in either group (**Table 3**). Both in the DS group and in the control group, HOMA-IR > 2.5 was observed in 38% of the study participants. In DS syndrome, an increased incidence of endocrine gland diseases is observed (26). Our study showed statistically significant differences in both TSH (p=0.009) and fT4 (<0.001) levels. If we compare the effect of age ≤ 18 and > 18 years on TSH and fT4, the significance for TSH was noted only in the DS group vs. control > 18 years of age (p=0.02), and fT4 was significant only in the group ≤ 18 years of age (p=0.008).

**Table 3:** Biochemical variables.

Variables	References value	Group with DS n=42	Control group n=21	p-value
		mean ± SD	mean ± SD	
TSH [uIU/ml]	0,4-4,2 mIU/l	1.5 ± 1.9	1.9 ± 0.7	0.009
fT4 [ng/dl]	0.8–1.7	0.7 ± 0.2	1.3 ± 0.1	<0.001
Glucose [mg/dl]	70-99	86.6 ± 8.7	86.4 ± 7.0	0.902
Insulin [uIU/ml]	< 10	10.6± 5.9	11.1 ± 5.5	0.922
HOMA -IR	< 2.5	2.3 ± 1.3	2.3 ± 1.4	0.748
HDL- C [mg/dl]	>35	47.0 ± 10.2	59.2 ± 9.6	<0.001
TC [mg/dl]	< 170	182.1 ± 3.4	173.6 ± 22.3	0.387
TG [mg/dl]	<100	97.6 ± 52.8	68.4 ± 21.4	0.056
LDL-C [mg/dl]	< 110	104.3 ± 25.1	89.8 ± 15.9	0.028
non-HDL [mg/dl]	<130	136.2 ± 35.7	109.0 + 26.5	0.022
Apo A1 [mg/dl]	108-225	119.6 ± 19.9	158.2 ± 31.4	<0.001
Apo B [mg/dl]	50-150	67.4 ± 16.6	61.1 ± 11.2	0.152
Homocysteina [umol/L]	<15	9.6 ± 2.5	10.2 ± 2.3	0.532
Lp (a) [nmol/l]	< 75	54.3 ± 80.2	51.0 ± 62.7	0.349
Albumin [g/L]	F 37-53 M- 42-55	36.7 ± 13.8	40.5 ± 4.5	0.325
Creatinine [mg/dL]	F 0.6-1,1 M 0.9-1,3	0.7 ± 0.3	0.7 ± 0.2	0.748
Urea [mg/dL]	10-50	26.3 ± 11.5	23.8 ± 5.9	0.086
LDH [U/L]	120-240	186.5 ± 109.1	171.7 ± 52.6	0.988

TSH, Thyroid-stimulating hormone; fT4, free thyroxine; HDL-C, high density lipoprotein level; TC, total cholesterol; TG, triglycerides, LDL-C, low density lipoprotein cholesterol, non –HDL, non high -density- lipoprotein, Apo A1, apolipoprotein A1, Apo B, apolipoprotein B, Lp (a), Liporotein a; LDH, lactate dehydrogenase; F, female; M, male.

### 3.4 Inflammatory variables

The analysis of inflammatory parameters did not show any statistically significant differences in the level of the strongly reactive acute phase protein CRP (p=0.677). However, the analysis of inflammatory status showed significant differences in cytokines levels. The anti-inflammatory IL-10 was significantly lower (p=0.006) in DS patients (21.2 ± 8.8) compared to healthy control (29.8 ± 8.6). Statistical significance between groups was also demonstrated in the levels of IL-5 (p=0.004) and IL-13 (p<0.001), where significantly lower values were recorded in DS (**Table 4**). The evaluation of IL-22, which has both pro- and anti-inflammatory roles depending on the specific context, tissue, and disease, was even two-fold lower in DS (p=0.002). A strong correlation was shown between IL-5 and IL-22 (rs= 0.946, p= < 2.2e-16), and also the correlation trend between IL-10 and IL-13 (rs=0.508 p<0.001). The comparative analysis of patients by age (≤ 18 years and > 18 years) showed the same significance as when patients were divided without taking into account age, therefore the observed changes in cytokines are not dependent on age.

**Table 4:** Inflammatory variables.

Variables	Group with DS n=42	Control group n=21	p-value
	Mean ± SD	Mean ± SD	
CRP [µg/mL]	0.1 ± 0.2	0.1 ± 0.1	0.677
IL-2 [pg/mL]	10.2 ± 5.6	9.7 ± 4.9	0.751
IL-5 [pg/mL]	10.8 ± 9.0	22.0 ± 16.2	0.004
IL-10 [pg/mL]	21.2 ± 8.8	29.8 ± 8.6	0.006
IL-13 [pg/mL]	100.3 ± 43.3	154.7 ± 38.7	<0.001
IL-22 [ng/L]	51.1 ± 42.1	108.3 ± 80.6	0.002
IL-33 [ng/L]	94.5 ± 39.8	84.2 ± 18.4	0.127

CRP, C reactive protein.

### 3.5 Assessment of specific variables in the analysis of the association between Down syndrome inflammation and immunometabolic status

The analysis of indicators in the immunometabolic status showed low AUC values for carbohydrate metabolism parameters: glucose = 0.487 (sens= 40%, spec. 50%) and for insulin = 0.509 (sens= 20.0%, spec. = 90.5%) (**Table 5**). For the lipid profile, the highest sens values were recorded for triglycerides = 61.9% and the highest spec. for apolipoprotein A = 95.2%, also for Apo A1 the highest AUC value was recorded = 0.818. The analysis of the ROC curves for cytokines demonstrated the high diagnostic potential for clinical prognosis in DS. The cut-off values for IL-5 and IL-10 were 9.67 and 29.86, respectively. The cut-off values for the remaining cytokines were: for IL-13 = 150.58 (AUC = 0.814), and for IL-22 = 47.55 (AUC=0.736). The highest specific sensitivity >80% was noted for IL-5 (80.9) and IL-22 (95.0). Specific sensitivity >80% was noted for IL-10 (85.7) and IL-13 (88.1).

**Table 5:** The statistical characteristics of the ROC curve for the univariate logistic model.

Variables	AUC	Cut-off value	Sensitivity (%)	Specificity (%)
Insulin [uIU/ml]	0.509	19.1	20.0	90.5
Glucose C [mg/dl]	0.487	88	40.0	50.0
LDL-C C [mg/dl]	0.629	106	42.1	89.5
TG [mg/dl]	0.652	77	61.9	70.0
Apo A1 [mg/dl]	0.818	144	60.0	95.2
TSH [uIU/ml]	0.713	1.93	74.3	73.7
fT4 [ng/dl]	0.777	1.28	94.8	21.0
IL-5 [pg/mL]	0.725	9.67	80.9	59.5
IL-10 [pg/mL]	0.749	29.86	57.1	85.7
IL-13 [pg/mL]	0.814	150.58	71.1	88.1
IL-22 [ng/L]	0.736	47.55	95.0	11.9

AUC, are under curves; LDL, low density lipoprotein; TG; triglycerides; TSH, Thyroid-stimulating hormone; fT4, free thyroxine; TG; triglycerides, Apo A1, apolipoprotein A1.

## 4 Discussion

In Down syndrome the interplay of inflammation, including increased activation of inflammatory cytokines and gene and metabolite expression, leads to coordinated regulation of metabolism (17). People with DS experience significant immune dysregulation, which makes them more susceptible to infections, especially upper respiratory tract infections, compared to people of the same age without DS (27). Inflammation is recognized as an independent/residual risk factor for cardiovascular and carbohydrate diseases. The interaction between these conditions, especially in individuals with DS, is less clear. Our study is one of the few in Poland to assess the metabolic status: carbohydrate and lipid metabolism in relation to inflammatory parameters in patients with DS compared to the control group consisting of siblings.

Obesity is estimated to be more common in individuals with DS in the general population (28, 29). It is difficult to clearly identify the causes of the observed disorders, but many authors (30, 31) believe that it may be a consequence of, firstly, reduced physical activity and poor eating habits, and secondly, co-occurring endocrine disorders in patients with Down syndrome. Our study did not show any significant differences in anthropometric indicators assessing body weight such as BMI (p=0. 241), BMI SDS (p=0.557). To more accurately assess the level of body fat in children and adolescents, the TMI index was introduced. The results showed statistical significance between the groups (p=0.04), which may indicate that the group of patients with DS may be more vulnerable to dangerous patterns of body fat distribution, indicating a greater health risk (17). Similarly, the study by Shim et al. (32) involving n= 8,464 subjects aged 10–20 years showed that the TMI, depending on gender and age, may be useful in clinical settings as a screening tool for overweight and obese children and adolescents. Endocrine disorders are often observed in conjunction with excess body fat. Thyroid hormone (TH) is involved in cholesterol metabolism and glucose homeostasis (33). Hypothyroidism can lead to lipolysis and reduced glucoconeogenesis, which in turn leads to weight gain (34). According to Amr (35), children with DS more often have TSH levels in the upper range of normal, and T4 levels in the lower range of normal. Comparisons of the entire group, without division by age, showed statistical significance for both TSH (p = 0.009) and fT4 (p <0.001), and ROC curve analysis showed relative diagnostic usefulness for both parameters: TSH - AUC = 0.713 (sens. 74.3% spec. 73.7%) and fT4 AUC = 0.777 (sens. 94.8% spec. 21.0%). However, the results should be interpreted with caution, as the low specificity for fT4 may indicate that this indicator may be useful only in the initial screening test. Analysis adjusted for age revealed no significant differences between TSH and the younger group (DS vs. control group) ≤ 18 years of age. Significance was demonstrated only when comparing the DS group vs. the control group > 18 years of age (p = 0.02). The observed discrepancies may be due to the longer duration of the disease and, consequently, the development and growth of patients may require a modification of the drug dose at some stage. In the DS group, 100% of patients were taking L-thyroxine. This high rate is a result of recruiting patients from an endocrinology clinic and the clinical practice of initiating L-thyroxine treatment early to support neurodevelopment in children with Down syndrome. We know that thyroid dysfunction and its treatment can affect lipid metabolism, glucose homeostasis (HOMA-IR), and cytokine profiles. However, when we examined the pediatric group (aged ≤ 18 years), TSH levels were well controlled and did not differ from those in the control group. Even with thyroid function controlled, these young patients still showed significant changes in lipid fractions and inflammatory parameters. This indicates that immunometabolic differences are likely due to the genetics of Down syndrome (trisomy 21) itself. Nevertheless, the high rate of L-thyroxine use in our cohort may influence the results, limiting its use in the general population with Down syndrome. There are reports that individuals/children with DS are also at an increased risk of developing metabolic disorders, including diabetes. However, the scientific literature often fails to distinguish between the different types of diabetes and does not provide a biochemical characterization of this disorder (36). In our study group with DS, mean HOMA -IR values were 2.3 ± 1.3, with mean insulin concentrations of 10.6 ± 5.9 and glucose concentrations of 86.6 ± 8.7 and what’s interesting similar results were observed the control group. A study conducted by Fonseca et al. (37) with n=15 adolescents with DS (8 males and 7 females) showed a lower mean HOMA-IR value (1.7 ± 1.0) compared to our study, but the authors, contrary to our findings, observed a correlation between overweight and obesity and insulin resistance. Furthermore, the AUC values for insulin = 0.509 in our research have poor diagnostic utility. In turn similarly to us, Magge et al. (38) assessed glucose and insulin levels between a child with DS (n= 35) and their siblings (n=33) and also did not show significant differences in both glucose (p=0.095) and insulin concentration (p = 0.3). The observed differences in present research may result from different sizes of the studied groups. It remains unclear whether individuals with DS exhibit a particularly atherogenic profile before developing obesity and diabetes. On the one hand, Down syndrome was historically considered “atherosclerosis-free” (12). A subsequent large epidemiological study by Day et al. (39) involving >14,000 individuals with DS in California (mean age at the start of follow-up was 14 years 8 months) showed a 4.3-fold higher mortality rate from coronary heart disease in DS. In our study, patients from the DS group showed higher concentrations of atherogenic lipoproteins: TG (97.6 ± 52.8; p=0.056) and LDL-C (104.3 ± 25.1; p=0.028) compared to the control group. Statistically significantly lower values were observed for HDL-C (p<0.001). The observations are consistent with the results of Zamorano et al. (40), where in the group of n= 72 children with DS and n= 66 healthy patients, similarly to us, they noted higher concentrations of both TG and LDL-C as well as TC and lower HDL-C in the group with DS. Interestingly, the concentration of another atherogenic fraction, Lp(a), was similar in both groups. It should be emphasized that the study included individuals of different ages, and that Lp(a) levels stabilize by approximately 5 years of age. It is also worth noting that Lp(a) concentrations are primarily influenced by genetics (41). The highest diagnostic usefulness in the case of lipids was observed for apolipoprotein A1 AUC = 0.818, and the decreased Apo A1 concentration in DS patients may be associated with a pro-inflammatory, insulin-resistant lipid phenotype. Given that the Down syndrome phenotype is associated with abnormal gene dosage on chromosome 21 and their subsequent overexpression, it is likely that changes in the lipid profile may result from genetic factors (42). This is confirmed by Bertapelli et al. which demonstrated higher cholesterol levels in fetuses with trisomy 21 during intrauterine development (43).

People with Down syndrome often have immunological disorders, which result in a poorer response to infection or greater susceptibility to autoimmune disorders (44). Unfortunately, there is still a paucity of studies assessing pro/anti-inflammatory cytokine levels in children with Down syndrome. IL-10 is a key cytokine that plays a fundamental role in modulating inflammation and maintaining cellular homeostasis. It acts primarily as an anti-inflammatory cytokine (45). During acute infection, IL-10 limits the scale of the immune response, protecting tissues from damage, but excessive production of this cytokine can lead to the inhibition of an effective immune response (45). In our study, we observed significantly higher IL-10 levels in the control group compared to the DS group (p=0.006), and relatively high diagnostic usefulness for this cytokine (AUC = 0.749). A study by Rostami et al. (46) involving three study groups (children with DS, healthy controls, and children with intellectual disability (ID) and they showed that serum from the ID group had significantly higher IL-10 levels compared with those from the DS group (p<0.05), but not the healthy, control group. The surprising differences in IL-10 levels between the groups in our study may be related to the fact that people with DS have a chronic low-grade inflammation: “baseline inflammation,” and anti-inflammatory regulation is weakened. This may also result in a shift in the immune balance towards pro-inflammatory. However, due to the small number of subjects, this does not allow us to draw clear conclusions. It should be emphasized that autoinflammation in Down syndrome is well documented, although it is not clear whether the disturbance of innate immunity causes this inflammation or vice versa (47). Research indicates that elevated Th2 cytokines like IL-4 and IL-13 in the plasma of DS individuals are key contributors to basal T-cell activation and pathogenic autoantibody production (48). Role of IL-13 was assessed by the authors of Nigam et al. (49) in a meta-analysis that included a total of n = 10 articles. In the case of IL-13, the authors showed higher levels in the DS group compared to the control group, but there was no statistical significance (Cohen’s d = 11.57, 95% CI = -17.95 to 41.09, p = 0.44). Interestingly, in our study we observed significantly higher levels of this cytokine in the control group compared to children with DS (p=0.002). In turn, the study conducted by Malle et al. (7) involving n=21 individuals with DS and n=10 controls showed that T2 cell cytokines: IL-4 and IL-13 were significantly elevated in the DS group. The discrepancy in the analyzed results may have various origins. On the one hand, it should be emphasized that the groups in both studies were similar. Malle et al. (7) recruited patients in the age range of 7 months - 37 years, which could suggest similar observations. On the other hand, the observed lower IL-13 results may result from weaker production of Th2 cytokines (including IL-13) in our study group, which should be assessed by performing further tests, including flow cytometry. It is also worth mentioning that the our group of children with DS has significantly higher differences in TMI indices, which may suggest that the coexisting presence of excessive adipose tissue and a changed phenotype of M2 to M1 macrophages cause a reduction in systemic IL-13 activity. The study by Meguid et al. (50) showed that serum IL-2 levels were significantly decreased in children with DS compared to the control group. Our study, unlike the study by another Polish group, Smielska-Kuzia et al. (51), did not show any significance in IL-2 levels (p=0.751), but similarly to the authors, we noted higher mean IL-2 levels in the DS group (10.2 ± 5.6) compared to the control group (9.7 ± 4.9). Studies conducted by Malle et al. (7) showed that the levels of proinflammatory cytokines IL-6, IL-1α, and TNFβ were significantly higher in patients with DS compared to the control group. However, the assessment of the proinflammatory cytokine IL-5 has not been widely reported. In our study, we demonstrated relatively high diagnostic usefulness of this cytokine (AUC = 0.725) and significantly higher values in the control group. In contrast, the study by Rostami et al. (46) did not show differences in IL-5 levels between the groups. There are not many studies that directly assess IL-5 in people with DS, which, on the one hand, indicates that IL-5 is not a central cytokine in the immunopathology of Down syndrome. Nevertheless, taking into account the function of IL-5, changes in its level may be associated with disturbed Th1/Th2 balance, but due to the small number of people studied, no clear conclusions can be drawn and further research is required. In patients with DS, activation of JAK-STAT signaling pathways leads to the formation of interferon regulatory factor (IRF) complexes. Consequently, total and phosphorylated Signal Transducer and Activator of Transcription 1 (STAT1) levels are constitutively higher in both unstimulated and interferon-stimulated monocytes of individuals with Down syndrome. We know also that IL-5 is one of the key cytokines responsible for eosinophil maturation. Therefore, the possible cause of the observed disproportion may be the presence of other diseases, including allergies. Explaining these observations requires continued research and verification of allergy tests. Consequently, increased expression of proinflammatory cytokines and chemokines that act downstream of interferon-induced signaling, such as L-22, CCL2, and Vascular Endothelial Growth Factor A (VEGFA), is observed in individuals with DS (52). Interestingly, our study observed significantly lower levels of IL-22 compared to the control group. Further research may be needed to explain these observations, but given that IL-22 is involved in maintaining the integrity of the mucosal barrier, the increased incidence of intestinal dysbiosis in individuals with DS may secondarily reduce stimulation of the Th17/ILC3 axis, affecting IL-22 levels.

Understanding Down syndrome as an immunometabolic disorder has important clinical implications. This highlights the need for integrated therapeutic strategies that target both inflammatory signaling and metabolic regulation. Unfortunately, there are still few studies assessing the immune profile using both pro- and anti-inflammatory cytokines. Further longitudinal and tissue-specific studies are needed to define age- and tissue-specific immunometabolic signatures in Down syndrome and to identify biomarkers and interventions that may mitigate immune dysfunction and improve long-term health outcomes in this population.

## 5 Conclusion

Our study identifies the role of inflammatory parameters in carbohydrate and lipid metabolism in individuals with Down syndrome—in children, adolescents, and adults. We demonstrated statistically significant changes in the levels of cytokines IL-5, IL-10, IL-13, and IL-22, which lead to immune dysfunction, suggesting a heterogeneous cytokine profile in the Down syndrome group. Higher TMI values in the Down syndrome group (≤ 18 years of age) are also associated with higher levels of proatherogenic lipids, i.e., non-HDL (p=0.02) and apoB (p=0.04), strengthening the idea that the coexistence of chronic inflammation and lipid metabolism disorders may correspond to an increased risk of atherosclerosis and other cardiometabolic disorders in individuals with an extra pair of chromosomes.

## 6 Limitation

Our study has several limitations: 1) the small number of study participants and the small control group size are not representative enough to draw firm conclusions. Further studies with a larger sample size are necessary; 2) L-thyroxine intake in DS group which may potentially affect lipid profile and immunological parameters; 3) The group of patients with Down’s syndrome studied had been under the care of an endocrinologist since infancy which may potentially affect lipid profile and immunological parameters.

## Data Availability

Datasets are available upon request: The raw data supporting the conclusions of this article will be made available by the authors, without undue reservation.
